# The efficacy of contrast-enhanced computed tomography on the management of gastroesophageal varices in patients with hepatocellular carcinoma

**DOI:** 10.1038/s41598-022-25350-8

**Published:** 2022-12-01

**Authors:** Takayuki Kondo, Kisako Fujiwara, Miyuki Nakagawa, Hidemi Unozawa, Terunao Iwanaga, Takafumi Sakuma, Naoto Fujita, Keisuke Koroki, Hiroaki Kanzaki, Kazufumi Kobayashi, Soichiro Kiyono, Masato Nakamura, Naoya Kanogawa, Tomoko Saito, Sadahisa Ogasawara, Eiichiro Suzuki, Yoshihiko Ooka, Shingo Nakamoto, Tetsuhiro Chiba, Makoto Arai, Jun Kato, Naoya Kato

**Affiliations:** 1 Department of Gastroenterology, Graduate School of Medicine, Chiba University, 1-8-1, Inohana, Chuo-Ku, Chiba, 260-8670 Japan; 2 Translational Research and Development Center, Chiba University Hospital, Chiba, Japan; 3 Department of Gastroenterology, Tokyo Women’s Medical University Yachiyo Medical Center, Chiba, Japan

**Keywords:** Gastroenterology, Hepatology

## Abstract

The screening of gastroesophageal varices (GEV) is critical in hepatocellular carcinoma (HCC) management. Contrast-enhanced computed tomography (CECT) is often performed in patients with HCC. Therefore, this study aimed to examine the use of CECT in screening for GEV and predicting GEV bleeding. This retrospective study enrolled 312 consecutive patients who are initially diagnosed with HCC, measured the lower esophageal (EIV) and fundal intramural vessel (FIV) diameter on CECT, examined the changes after 1, 2, and 3 years, and verified the relationship with GEV bleeding. The EIV and FIV diameter on CECT correlates well with endoscopic variceal classification. EIV significantly worsened after 2 and 3 years. FIV showed worsening at both 1, 2, and 3 years. Cumulative GEV bleeding rates were 3.7% at 1 year and 6.2% at 3 years. The multivariate analysis revealed that EIV, FIV, and portal vein tumor thrombus were associated with GEV bleeding. Furthermore, EIV deterioration at 1, 2, and 3 years correlated with GEV bleeding. In conclusion, CECT is useful in variceal management during the longitudinal clinical course of HCC, and has the potential to decrease screening endoscopy. With deterioration in EIV, treatments should be considered due to a high-risk GEV bleeding.

## Introduction

The prevalence of gastroesophageal varices (GEV) is almost half in patients with cirrhosis, although it varies depending on the clinical stage^1,2^. Further, GEV bleeding is one of the major complications of cirrhosis and heralds their poor prognosis despite significant GEV management improvements^3–5^. A recent study reported that the prevalence of portosystemic shunt is 60% in patients with cirrhosis and increases the risk of complications and deaths^6^.

Hepatocellular carcinoma (HCC) remains one of the leading causes of cancer-related death worldwide^7,8^. Thus, a proper assessment of disease severity, treatment, and surveillance is necessary to improve the prognosis in patients with HCC^8,9^. Previous studies demonstrate that GEV is concomitant with > 50% of HCC, and GEV screening is critical in HCC management^10–12^. The American Association for the Study of Liver Diseases (AASLD) and the European Association for the Study of the Liver guidelines dictate how to perform screening esophagogastroduodenoscopy (EGD), depending on the presence or absence of cirrhosis, decompensation history, or esophageal varices (EV)^13,14^. However, in patients with HCC, the exact interval for screening EGD is unclear, and performing the endoscopic screening annually in an overcrowded treatment schedule is inefficient and not cost effective. Contrast-enhanced computed tomography (CECT) is often performed for HCC screening and treatment response assessment on HCC, and the ability of CECT to directly visualize EV has been reported^15–17^. However, its ability to visualize gastric varices and manage GEV on the longitudinal clinical course of HCC remains unclear.

Therefore, the current study aimed to examine the use of CECT in screening GEV and predicting GEV bleeding during the long clinical course of HCC.

## Patients and methods

### Patients

This retrospective study enrolled consecutive patients who are initially diagnosed with HCC between 2011 and 2014, measured the maximal short-axis diameter of lower esophageal (EIV) and fundal intramural vessels (FIV) on CECT, examined the changes between the time of diagnosis and 1, 2, and 3 years later, and verified the relationship with variceal bleeding. Further, CECT and endoscopic findings in patients who underwent EGD within 3 months from CECT were compared. This study set out the following exclusion criteria: (i) patients with HCC who were not diagnosed by CECT; (ii) patients with a history of stomach or esophageal surgery; (iii) patients with advanced cancer other than HCC; and (iv) patients with esophageal achalasia.

CECT was performed using a 64-detector CT scanner (Aquilion 64, Toshiba), an 80-detector CT scanner (Aquilion prime), or a 320-detector CT scanner (Aquilion ONE, Toshiba). The contrast agent was used at a dose of 100 mL and an infusion rate of 3 mL/s by mechanical injection via a peripheral vein. Images were taken in three phases as follows: hepatic arterial, portal venous, and equilibrium. Esophageal and fundal varices were defined as intramural enhancing nodular tubular structures that protrude into the esophageal and fundal lumen or run adjacent to the inner esophageal and fundal mucosa, using a 5-mm slice thickness CECT of the portal venous phase^17^. The portosystemic shunt was considered as spontaneous communications between portal circulation and the systemic venous system, excluding GEV^6^.

### Definitions

HCC was diagnosed according to the diagnostic criteria by the AASLD and is stratified into early-stage (single of any size or ≤ 3 nodules of ≤ 3 cm in diameter), intermediate-stage (> 3 nodules of any size or 2–3 nodules of > 3 cm in diameter), and advanced-stage (any nodules with macrovascular invasion or extrahepatic spread) groups. The treatment strategy for HCC was discussed at a multidisciplinary meeting. After explaining the advantages and side effects of various therapies and recommendations from the experts, patients finalized the treatment strategy. Potentially curative treatment was defined as surgical resection or ablation^18^. Cirrhosis was defined according to a combination of clinical signs and findings provided by laboratory tests, radiologic imaging, or liver biopsy. Hepatic encephalopathy (HE) was assessed using the West Haven grading system^19^. The degree of ascites was defined according to the international guidelines as follows^20^: mild, ascites that were only detectable by ultrasound examination; moderate, ascites that caused moderate symmetrical abdominal distension; and severe, ascites that caused marked abdominal distension. The diagnosis of spontaneous bacterial peritonitis was confirmed with an ascitic neutrophil count of > 250 cells/mm^3^ with no intra-abdominal and surgically treatable source of sepsis^20^.

### Endpoint

The primary endpoint was the occurrence of variceal bleeding. The key secondary endpoint was overall survival, which covered the time from the date of enrollment to the date of death, the last visit, or loss to follow-up.

### Statistical analysis

All data are expressed as the mean ± standard deviation or percentage. Continuous variables were analyzed using Student’s *t*-test, the Mann–Whitney *U*-test, or the paired *t*-test as appropriate. Categorical variables were analyzed using Fisher’s exact test or the chi-squared test, as appropriate. The cumulative survival rate was calculated using the Kaplan–Meier method. The risk factors for GEV bleeding were evaluated by Cox regression analysis. The best cutoff value was calculated according to the area under the receiver operating characteristics curve (AUC) analysis. Further, a *p* value of < 0.05 was considered significant, and statistical data were analyzed using SAS version 9.2 (SAS Institute, Cary, NC).

### Ethical approval

This study does not contain animal experimental data. This study conformed to the principles of the Declaration of Helsinki and was approved by the Ethics Committee of Chiba University Graduate School of Medicine.

## Results

### Patient characteristics and CT findings compared to endoscopic findings

**Figure 1:**
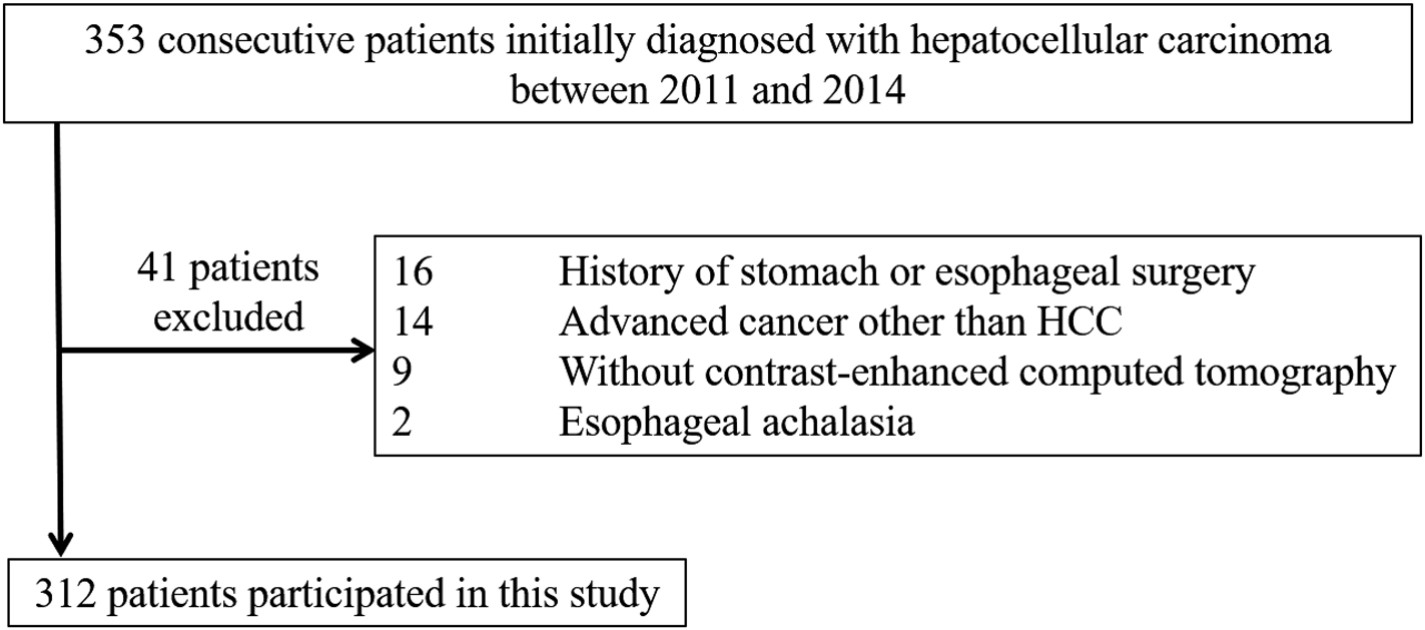
Protocol diagram.

This study included 312 subjects (Table 1). The study flow chart is shown in Fig. 1. The median observation period was 39.7 months. Initially, 179 (57.4%) patients received potentially curative treatment for HCC. The cumulative overall survival rate was significantly higher in the curative treatment group (n = 179, 96.0%, 91.8%, and 85.0% at 1, 2, and 3 years, respectively) than in the noncurative treatment group (n = 133, 62.0%, 41.8%, and 33.8% at 1, 2, and 3 years, respectively; *p* < 0.001).

**Table 1:** Patient characteristics.

Number of patients	312
Age (years)	68.8 ± 9.6
Sex (male/female)	209/103
Liver cirrhosis	214 (68.6%)
HCC early/intermediate/advanced stage	202/59/51
Prior history of acute decompensation	76 (24.4%)
Potentially curative treatment for HCC	179 (57.4%)
Prior history of variceal bleeding	13 (4.2%)
Prior history of treatment for gastroesophageal varices	19 (6.1%)
Etiology (virus/alcohol /NASH/PBC/AIH/others)	196/39/18/7/3/49
Child–Pugh A/B/C	259/51/2
Albumin-bilirubin (ALBI) score	− 2.34 ± 0.51
Model for end-stage liver disease score	7.1 ± 3.2

*AIH* autoimmune hepatitis, *HCC* hepatocellular carcinoma, *NASH* nonalcoholic steatohepatitis, *PBC* primary biliary cholangitis; potentially curative treatment for HCC: liver resection or ablation.

Among the 312 consecutive patients, 231 patients underwent EGD within 3 months before and after CECT. The EIV diameter, subdivided based endoscopic variceal classification, was as follow: no varices. 1.1 ± 1.3 mm (n = 165); small varices (F1), 4.1 ± 0.8 mm (n = 41); medium varices (F2), 7.3 ± 1.2 mm (n = 23); and large varices (F3), 9.2 ± 3.0 mm (n = 2). The best cutoff values were F1: 3.1 mm (AUC = 0.986) and F2: 5.5 mm (AUC = 0.995). There was no red-color (RC) sign on EGD in the no varices group, and 7 (17.1%) patients in the F1 EV group and 10 (40.0%) patients in the F2-3 EV group exhibited the RC sign. The best cutoff value for the RC sign was 4 mm of the EIV diameter (AUC = 0.936).

The FIV diameter, subdivided based on endoscopic variceal classification, was as follows: no varices, 0.1 ± 0.5 mm (n = 210); small varices (F1), 3.7 ± 1.5 mm (n = 10); medium varices (F2), 8.7 ± 2.1 mm (n = 5); and large varices (F3), 12.0 ± 2.4 mm (n = 6). The best cutoff values were F1:3.4 mm (AUC = 0.972) and F2: 5.7 mm (AUC = 0.999).

The cumulative overall survival rate was significantly lower in patients with EV of F1 or greater on CECT (n = 84, 78.2%, 59.9%, and 54.4% at 1, 2, and 3 years, respectively) than those without (n = 228, 83.4%, 76.0%, and 68.2% at 1, 2, and 3 years, respectively; *p* < 0.001).

### GEV bleeding

**Figure 2:**
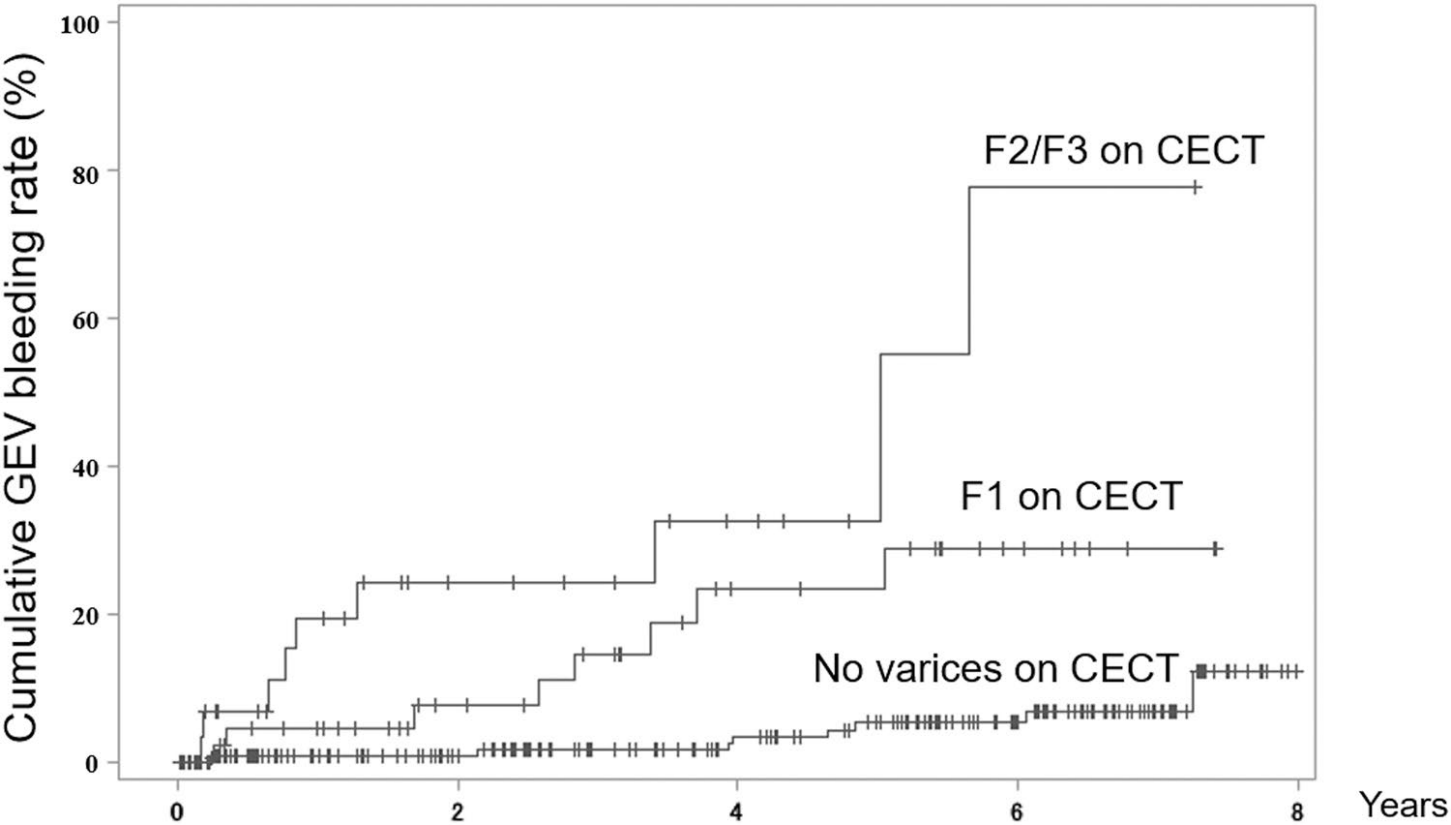
Comparison of cumulative GEV bleeding rates between no varices, F1, and F2/3 on CECT. CECT: contrast-enhanced computed tomography; F1: small varices; F2/3: medium to large varices; GEV: gastroesophageal varices.

During the study periods, 26 patients had GEV bleeding and 7 received prophylactic EV treatment. Cumulative GEV bleeding rates were 3.4%, 5.9%, and 10.8% at 1, 3, and 5 years, respectively. Table 2 shows the predictive factors for GEV bleeding according to the univariate analysis. The multivariate analysis revealed the EIV diameter (*p* < 0.001), FIV diameter (*p* = 0.011), and the presence of portal vein tumor thrombus (PVTT) (*p* = 0.043) as the significant predictive factors for GEV bleeding (Table 2). Cumulative GEV bleeding rates were significantly worsened with the severity of EV classification using CECT (no varices, F1, and F2–F3 of 1.0%, 4.6%, and 19.6% at 1 year; 1.0%, 7.6%, and 24.3% at 2 years; 1.6%, 14.7%, and 24.3% at 3 years, respectively, *p* < 0.001, Fig. 2). No significant difference was found in GEV bleeding between HCC stages. However, the 1-year cumulative GEV bleeding rate was significantly higher in patients with advanced HCC stages (10.7%) than in those with early-intermediate HCC stages (2.5%, *p* = 0.034). The cumulative overall survival rate was significantly lower in patients with variceal bleeding within 1 year after HCC diagnosis (55.6%, 33.3%, and 33.3% at 1, 2, and 3 years, respectively) than in those without (82.8%, 72.8%, and 65.5% at 1, 2, and 3 years, respectively; *p* = 0.013).

**Table 2:** Cox regression analyses of predictive factors for variceal bleeding.

	Univariate hazard ratio (95% confidence interval)	*p* values	Multivariate hazard ratio (95% confidence interval)	*p* values
Age	0.964 (0.928–1.001)	0.059	–	
Male sex	1.533 (0.644–3.650)	0.334	–	
Liver cirrhosis	7.246 (1.708–30.742)	0.007	–	
Prior history of acute decompensation	5.931 (2.602–13.520)	< 0.001	–	
Ascites	2.147 (0.944–4.882)	0.068	–	
Intermediate-advanced HCC	1.764 (0.716–4.345)	0.217	–	
Portal vein tumor thrombosis	3.703 (1.078–12.716)	0.038	3.726 (1.045–13.289)	0.043
Potentially curative treatment for HCC	0.580 (0.251–1.338)	0.202	–	
Prior history of variceal bleeding	0.000	0.988	–	
Prior history of treatment for gastroesophageal varices	0.000	0.989	–	
Alcohol related hepatitis	2.336 (0.936–5.834)	0.069	–	
**Findings on contrast enhanced CT**				
Diameter of intramural vessel in esophagus	1.543 (1.334–1.784)	< 0.001	1.532 (1.316–1.784)	< 0.001
Diameter of intramural vessel in fundus	1.182 (1.068–1.307)	0.001	1.141 (1.030–1.264)	0.011
Diameter of portosystemic shunt	1.007 (0.920–1.102)	0.882	–	
**Laboratory data**				
Alanine aminotransferases (U/L)	1.005 (0.997–1.013)	0.223	–	
Bilirubin (mg/dL)	1.323 (1.027–1.705)	0.030	–	
Prothrombin time (international normalized ratio)	2.201 (0.196–24.722)	0.523	–	
Albumin (g/dL)	0.348 (0.153–0.792)	0.012	–	
Creatinine (mg/dL)	0.807 (0.370–1.758)	0.589	–	
Platelets (10^9^/L)	0.943 (0.880–1.011)	0.943	–	
Alfa fetoprotein (ng/ml)	1.000 (1.000–1.000)	0.238	–	
Albumin-bilirubin (ALBI) score	3.624 (1.597–8.225)	0.002	–	
Child–Pugh score	1.723 (1.141–2.601)	0.010	–	
Model for end-stage liver disease (MELD) score	1.006 (0.872–1.161)	0.933	–	

*CT* computed tomography, *HCC* hepatocellular carcinoma.

Of the 196 patients with viral hepatitis, 91 (46.4%) received antiviral therapy. The cumulative GEV bleeding rates tended to be lower in patients who received antiviral therapy (n = 91, 1.1%, 1.1%, and 1.1% at 1, 2, and 3 years, respectively) than those who did not (n = 105, 2.6%, 4.0%, and 4.0% at 1, 2, and 3 years, respectively, *p* = 0.051).

Among the 231 patients who underwent EGD, the RC sign (relative risk = 5.035, 95% confidence interval 2.245–11.293) and F1 or more in the EV classification using CECT (relative risk = 9.775, 95% confidence interval 3.412–28.001) were associated with GEV bleeding.

### Changes in gastroesophageal intramural vessels and portosystemic shunt over time

CECT was conducted in 157, 141, and 131 patients after 1, 2, and 3 years, respectively. The EIV diameter did not significantly change after 1 year, but the FIV diameter and portosystemic shunt significantly worsened after 1 year (Table 3). The EIV and FIV diameter and portosystemic shunt significantly deteriorated after 2 and 3 years (Table 4). When divided according to the presence or absence of EV based on the classification using CECT, no significant change was found in the EIV diameters in patients with 3 mm or lesser EIV diameters, but an FIV diameter and portosystemic shunt worsening were observed. In the case of patients without cirrhosis, there is only 1 patient with EIV diameter > 3 mm and no patient with FIV diameter > 3.3 mm, and no significant change was found in EIV and FIV diameter and portosystemic shunt over time. The presence of fundal varices based on the classification using CECT and large portosystemic shunt has a positive impact on esophageal variceal exacerbations over time (Tables 3 and 4). When stratified according to HCC stage or treatment, a trend toward worsening the EIV and FIV diameter in the early stage and the curative treatment group was observed after 2 and 3 years (Tables 3 and 4).

**Table 3:** Change in diameter of variceal veins and portosystemic shunt after 1 year.

	Pre	1 year later	*p* values
**Diameter of vessel in esophagus (mm)**			
Overall (N = 157)	2.0 ± 2.1	2.2 ± 2.4	0.17
EIV ≤ 3 (N = 117)/ EIV > 3 (N = 40)	1.0 ± 1.1/4.9 ± 1.7	1.1 ± 1.3/5.3 ± 2.1	0.68/0.06
FIV ≤ 3.3 (N = 143)/ FIV > 3.3 (N = 14)	2.0 ± 2.2/2.5 ± 1.6	2.1 ± 2.5/2.5 ± 1.4	0.14/0.99
Non cirrhosis (N = 45)/cirrhosis (N = 112)	1.1 ± 1.2/2.4 ± 2.3	1.1 ± 1.4/2.6 ± 2.6	0.94/0.08
Early (N = 116)/Inter (N = 30)/Advanced stage (N = 11)	2.1 ± 2.2/1.9 ± 2.3/1.8 ± 1.5	2.2 ± 2.4/2.2 ± 2.7/2.0 ± 1.7	0.37/0.36/0.08
Potentially curative treatment (N = 104)/Noncurative treatment for HCC (N = 53)	2.0 ± 2.2/2.0 ± 2.1	2.2 ± 2.5/2.0 ± 2.4	0.08/0.87
**Diameter of vessel in fundus (mm)**			
Overall (N = 157)	0.6 ± 2.0	0.7 ± 2.2	0.05
EIV ≤ 3 (N = 117)/ EIV > 3 (N = 40)	0.5 ± 1.9/0.8 ± 2.4	0.7 ± 2.2/0.8 ± 2.3	0.03/0.77
FIV ≤ 3.3 (N = 143)/ FIV > 3.3 (N = 14)	0.04 ± 0.4/6.4 ± 2.9	0.1 ± 0.6/6.9 ± 3.3	0.17/0.12
Non cirrhosis (N = 45)/cirrhosis (N = 112)	0.1 ± 0.6/0.8 ± 2.4	0.2 ± 1.0/0.9 ± 2.5	0.31/0.07
Early (N = 116)/Inter (N = 30)/Advanced stage (N = 11)	0.7 ± 2.2/0.5 ± 1.0/0	0.8 ± 2.4/0.6 ± 2.0/0	0.06/0.23/–
Potentially curative treatment (N = 104)/Noncurative treatment for HCC (N = 53)	0.5 ± 1.9/0.8 ± 2.3	0.6 ± 2.1/0.8 ± 2.5	0.07/0.28
**Diameter of portosystemic shunt (mm)**			
Overall (N = 157)	3.1 ± 4.4	3.5 ± 4.7	< 0.01
EIV ≤ 3 (N = 117)/ EIV > 3 (N = 40)	2.6 ± 3.9/4.8 ± 2.5	2.9 ± 4.4/5.1 ± 5.0	< 0.01/0.33
FIV ≤ 3.3 (N = 143)/ FIV > 3.3 (N = 14)	3.1 ± 4.5/3.5 ± 4.7	3.4 ± 4.0/3.6 ± 4.0	< 0.01/0.51
Non cirrhosis (N = 45)/cirrhosis (N = 112)	0.4 ± 1.4/4.2 ± 4.7	0.5 ± 1.4/4.7 ± 4.9	0.40/ < 0.01
Early (N = 116)/Inter (N = 30)/Advanced stage (N = 11)	3.3 ± 4.7/3.5 ± 5.3/0.8 ± 2.1	3.6 ± 4.5/3.8 ± 5.5/1.3 ± 2.4	0.01/0.12/0.33
Potentially curative treatment (N = 104)/Noncurative treatment for HCC (N = 53)	3.0 ± 4.4/3.4 ± 4.6	3.4 ± 4.4/3.7 ± 4.7	0.01/0.04

Portosystemic shunt: maximum diameter of portosystemic shunt other than gastroesophageal varices.

*EIV* esophageal intramural vessel, *FIV* fundal intramural vessel.

Data are expressed as mean ± SD.

**Table 4:** Change in diameter of variceal veins and portosystemic shunt after 2 and 3 years.

	Pre	2 years later	*p* values
**Diameter of vessel in esophagus (mm)**			
Overall (N = 141)	1.8 ± 2.0	2.1 ± 2.8	0.03
EIV ≤ 3 (N = 112)/ EIV > 3 (N = 29)	1.0 ± 1.1/4.8 ± 1.6	1.2 ± 1.9/5.9 ± 2.6	0.39/ < 0.01
FIV ≤ 3.3 (N = 129)/ FIV > 3.3 (N = 12)	1.7 ± 2.0/2.5 ± 1.7	2.1 ± 2.9/2.5 ± 2.0	0.02/0.94
Non cirrhosis (N = 45)/cirrhosis (N = 96)	1.0 ± 1.2/2.2 ± 2.1	0.9 ± 2.1/2.7 ± 3.0	0.89/ < 0.01
Early (N = 114)/Inter (N = 18)/Advanced stage (N = 9)	1.7 ± 1.9/2.3 ± 2.7/1.5 ± 1.4	2.0 ± 2.7/2.8 ± 4.0/2.0 ± 1.7	0.07/0.29/0.42
Potentially curative treatment (N = 103)/Noncurative treatment for HCC (N = 38)	1.8 ± 1.9/1.6 ± 2.2	2.2 ± 2.8/1.8 ± 1.9	0.04/0.37
**Diameter of vessel in fundus (mm)**			
Overall (N = 141)	0.6 ± 2.1	0.8 ± 2.6	0.01
EIV ≤ 3 (N = 112)/ EIV > 3 (N = 29)	0.5 ± 1.9/1.0 ± 1.4	0.7 ± 2.3/1.4 ± 3.3	0.04/0.14
FIV ≤ 3.3 (N = 129)/ FIV > 3.3 (N = 12)	0.02 ± 0.2/6.9 ± 7.8	0.2 ± 1.0/7.8 ± 3.8	0.04/0.17
Non cirrhosis (N = 45)/cirrhosis (N = 96)	0.1 ± 0.6/0.9 ± 2.5	0.2 ± 0.7/1.2 ± 3.1	0.34/0.02
Early (N = 114)/Inter (N = 18)/Advanced stage (N = 9)	0.6 ± 2.2/0.9 ± 2.4/0	0.9 ± 2.7/0.8 ± 2.0/0	0.01/0.78/-
Potentially curative treatment (N = 103)/Noncurative treatment for HCC (N = 38)	0.6 ± 2.1/0.8 ± 2.2	0.9 ± 2.7/0.8 ± 2.2	0.01/0.80
**Diameter of portosystemic shunt (mm)**			
Overall (N = 141)	3.0 ± 4.3	3.6 ± 4.9	< 0.01
EIV ≤ 3 (N = 112)/ EIV > 3 (N = 29)	2.6 ± 3.8/4.9 ± 5.3	3.1 ± 4.5/5.7 ± 5.8	< 0.01/0.11
FIV ≤ 3.3 (N = 129)/ FIV > 3.3 (N = 12)	3.0 ± 4.3/3.1 ± 4.2	3.6 ± 4.9/3.5 ± 4.5	< 0.01/0.22
Non cirrhosis (N = 45)/cirrhosis (N = 96)	0.5 ± 1.4/4.3 ± 4.6	0.7 ± 1.8/5.1 ± 5.2	0.08/ < 0.01
Early (N = 114)/Inter (N = 18)/Advanced stage (N = 9)	3.1 ± 4.1/4.1 ± 4.1/0.7 ± 2.2	3.6 ± 4.7/5.2 ± 6.3/1.1 ± 2.2	< 0.01/0.05/0.47
Potentially curative treatment (N = 103)/Noncurative treatment for HCC (N = 38)	2.9 ± 2.8/3.4 ± 4.6	3.4 ± 4.8/4.4 ± 5.1	0.01/ < 0.01

	Pre	3 years later	*p* values
**Diameter of vessel in esophagus (mm)**			
Overall (N = 131)	1.7 ± 2.0	2.1 ± 2.7	0.01
EIV ≤ 3 (N = 105)/ EIV > 3 (N = 26)	0.9 ± 1.1/4.8 ± 1.6	1.2 ± 1.8/5.7 ± 3.0	0.13/0.03
FIV ≤ 3.3 (N = 122)/ FIV > 3.3 (N = 9)	1.6 ± 2.0/2.5 ± 1.9	2.0 ± 2.8/2.4 ± 1.8	0.01/0.78
Non cirrhosis (N = 45)/cirrhosis (N = 86)	0.9 ± 1.2/2.1 ± 2.2	0.8 ± 1.4/2.7 ± 3.1	0.76/ < 0.01
Early (N = 112)/Inter (N = 13)/Advanced stage (N = 6)	1.5 ± 1.8/2.9 ± 2.9/1.5 ± 1.8	1.9 ± 2.4/3.5 ± 4.8/2.8 ± 2.4	0.04/0.38/0.30
Potentially curative treatment (N = 102)/Noncurative treatment for HCC (N = 29)	1.6 ± 1.8/2.1 ± 2.3	1.9 ± 2.4/2.6 ± 3.6	0.02/0.26
**Diameter of vessel in fundus (mm)**			
Overall (N = 131)	0.5 ± 2.0	0.8 ± 2.6	< 0.01
EIV ≤ 3 (N = 105)/EIV > 3 (N = 26)	0.4 ± 1.8/0.9 ± 2.7	0.9 ± 2.7/1.1 ± 2.9	0.01/0.23
FIV ≤ 3.3 (N = 122)/FIV > 3.3 (N = 9)	0/7.4 ± 3.2	0.2 ± 1.0/8.9 ± 3.2	0.03/0.05
Non cirrhosis (N = 45)/cirrhosis (N = 86)	0.1 ± 0.6/0.7 ± 2.5	0.4 ± 1.4/1.1 ± 3.0	0.12/0.01
Early (N = 112)/Inter (N = 13)/Advanced stage (N = 6)	0.5 ± 2.0/0.7 ± 2.6/0	0.8 ± 2.6/1.0 ± 2.6/0	< 0.01/0.50/-
Potentially curative treatment (N = 102)/Noncurative treatment for HCC (N = 29)	0.5 ± 2.1/0.5 ± 1.9	0.8 ± 2.7/0.8 ± 2.1	0.01/0.20
**Diameter of portosystemic shunt (mm)**			
Overall (N = 131)	2.8 ± 4.0	3.5 ± 4.6	< 0.01
EIV ≤ 3 (N = 105)/ EIV > 3 (N = 26)	2.3 ± 3.4/4.8 ± 5.4	3.1 ± 4.2/5.3 ± 5.6	< 0.01/0.45
FIV ≤ 3.3 (N = 122)/ FIV > 3.3 (N = 9)	2.8 ± 3.9/2.9 ± 4.6	3.5 ± 4.6/3.3 ± 5.1	< 0.01/0.33
Non cirrhosis (N = 45)/cirrhosis (N = 86)	0.4 ± 1.3/4.1 ± 4.3	0.6 ± 1.8/5.1 ± 4.9	0.11/0.01
Early (N = 112)/Inter (N = 13)/Advanced stage (N = 6)	2.7 ± 3.8/4.1 ± 5.6/1.1 ± 2.7	3.3 ± 4.4/5.8 ± 6.3/2.0 ± 3.1	< 0.01/0.05/0.42
Potentially curative treatment (N = 102)/Noncurative treatment for HCC (N = 29)	2.8 ± 3.9/3.0 ± 4.3	3.3 ± 4.5/4.2 ± 5.0	0.01/0.01

Portosystemic shunt: maximum diameter of portosystemic shunt other than gastroesophageal varices.

*EIV* esophageal intramural vessel, *FIV* fundal intramural vessel.

Data are expressed as mean ± SD.

Focusing specifically on patients with viral hepatitis, antiviral therapy potentially slowed the exacerbation of the EIV (pre vs. 1 year later: with antiviral therapy, n = 54, 1.3 ± 1.5 vs. 1.3 ± 1.6 mm, *p* = 0.876; without antiviral therapy, n = 48, 3.0 ± 2.6 vs. 3.3 ± 3.0 mm, *p* = 0.022) and FIV diameter (pre vs. 1 year later: with antiviral therapy, 0.2 ± 1.1 vs. 0.3 ± 1.4 mm, *p* = 0.294; without antiviral therapy, 0.6 ± 2.0 vs. 0.7 ± 2.2 mm, *p* = 0.045) and portosystemic shunt (pre vs. 1 year later: with antiviral therapy, 2.5 ± 4.0 vs. 2.7 ± 3.9 mm, *p* = 0.156; without antiviral therapy, 3.1 ± 4.2 vs. 3.7 ± 4.6 mm, *p* = 0.002) after 1 year.

The EIV deterioration after 1, 2, and 3 years was significantly higher in patients with variceal bleeding (1.7 ± 1.2 mm, 2.9 ± 2.7 mm, and 3.8 ± 1.6 mm after 1, 2, and 3 years, respectively) than those without (0.1 ± 1.1 mm [*p* < 0.001], 0.2 ± 1.7 mm [*p* < 0.001], 0.1 ± 1.4 mm [*p* < 0.001] after 1, 2, and 3 years, respectively).

The best cutoff values were 0.3, 0.7, and 2.3 mm at 1, 2, and 3 years, respectively, (AUC = 0.883, 0.842, 0.961, respectively) correlated with variceal bleeding.

## Discussion

Several studies have evaluated the correlation between CECT and endoscopic findings of EV^15–17^. To our best knowledge, no studies have examined the findings of gastric varices on CECT and the efficacy of CECT on GEV management in patients with HCC. This study demonstrated the usefulness of CECT in visualizing GEV and the efficacy of CECT in GEV management during a long clinical course of HCC. The lower EIV and FIV diameter on CECT correlates well with endoscopic findings and variceal bleeding. Furthermore, lower EIV diameter deterioration on CECT during the long clinical course of HCC was associated with variceal bleeding. Additionally, the portosystemic shunt is likely to worsen within a year, and attention should be paid to the development of HE^6^.

EGD has been known as a gold standard in evaluating GEV. However, it is invasive, costly, and often requires sedation. Therefore, AASLD suggested that patients with a liver stiffness (LS) of < 20 kPa and a platelet count of > 150,000/mm^3^ have a very low probability (< 5%) of having high-risk varices, and screening EGD can be circumvented^13^. However, LS examination is often challenging in patients with HCC because of HCC or ascites. CECT is one of the most commonly used imaging modalities to perform a diagnosis of HCC and stage it, although it has its disadvantages, such as radiation exposure, contrast allergy, and kidney injury. The cutoff value for EV on CECT in this study was 3.1 mm for F1 and 5.5 mm for F2. This is consistent with previous reports with values of 5 mm or more, indicating a high-risk of variceal bleeding and values of < 3 mm, indicating a low risk of variceal bleeding^17^, as well as reports a 4-mm cutoff value for EV^15,16^. This study suggests that EGD can be reduced when varices are absent on CECT. In this study, 126 subjects showed no EIV on CECT, which may reduce EGD in approximately one-third of patients with HCC. Another advantage of CECT is the possibility of more variceal objective evaluation. The problem with endoscopic variceal evaluation is the changing appearance of varices depending on the amount of injected air.

The involvement of PVTT, in addition to GEV development, was a factor in variceal hemorrhage. Additionally, the advanced HCC stage was correlated with cumulative variceal bleeding at 1 year. However, the advanced HCC stage was not associated with the overall cumulative variceal bleeding due to the small number of patients and the short prognosis with advanced HCC stages, which may not be suitable for this long-term study. Further validation with larger cohorts is necessary, focusing on patients with advanced HCC, especially during chemotherapy. Furthermore, we found a correlation between the factors related to HCC and the deterioration of the EIV and FIV diameter. Interestingly, the early stage of HCC and potentially curative treatment negatively impacted the development of GEV. In the present study, 38.0% of patients in the curative treatment group received liver resection, unlike the noncurative treatment group, which did not include surgery. More patients in the early stage of HCC had undergone liver resection (27.2%) compared to 12.7% in the intermediate-advanced stage of HCC (*p* = 0.003). As previously demonstrated, liver resection causes worsened portal hypertension^21,22^. This simple but reasonable explanation may account for the deterioration of GEV in the early stage of HCC and the patients with HCC who received potentially curative treatment. However, further study may be required to unveil the effects of liver resection on GEV development.

Our study had several limitations. First, the data were retrospectively analyzed. Second, the RC sign is widely known as a factor in variceal hemorrhage^23–25^, but evaluating the RC sign by CECT is impossible. However, this study showed that the variceal bleeding rate could be stratified by dividing it by the F1 and F2 morphology on CECT, and the presence of F1 or more on CECT indicated sufficiently high relative risk comparable with RC sign. Furthermore, one study reported that all patients with CT varices of 4 mm or larger had RC signs on EGD^26^. Furthermore, we also found the best cutoff value for RC sign to be 4 mm of the EIV diameter on CECT. Another reported a correlation between EV size on CECT and the presence and severity of RC signs on endoscopic findings^16,27^. In this study, the RC sign was not observed in cases with less than F1 on CECT. Thirdly, this study did not assess cardia varices, which are characterized by a continuation of EV and were classified as type 1 GEV by Sarin et al.^28^. However, cardia varices are closely associated with the advanced grade of EV^25^. Therefore, lower EV evaluation may lead to cardia varices evaluation. Finally, although CECT is less invasive, the radiation exposure and possibility of renal dysfunction and allergic reactions makes evaluation of all patients and frequent imaging difficult. Therefore, our study focused on patients with HCC as they undergo frequent CECT evaluations. A previous study showed the usefulness of the EVendo score^29^, a machine learning-based scoring system using noninvasive data to screen high-risk patients with EV to avoid unnecessary EGD. Therefore, further studies are warranted to determine whether the variceal management of CECT is useful in patients without HCC, and whether there is an additive effect of CECT compared with noninvasive scores such as the EVendo score.

In conclusion, CECT is very useful in variceal management during the clinical course of HCC and could be employed in the evaluation of GEV. CECT has the potential to decrease EGD screening. However, if CECT cannot be performed due to renal dysfunction or contrast allergy, EGD is necessary within a year or two, especially with EV or cirrhosis.

## Data Availability

We made all data underlying the findings in this manuscript fully available.

## Abbreviations

AASLD: American association for the study of liver diseases
AUC: Area under the receiver operating characteristics curve
CECT: Contrast-enhanced computed tomography
EGD: Esophagogastroduodenoscopy
EIV: Esophageal intramural vessel
EV: Esophageal varices
FIV: Fundal intramural vessel
GEV: Gastroesophageal varices
HCC: Hepatocellular carcinoma
HE: Hepatic encephalopathy
LS: Liver stiffness
